# Orbital Reconstruction Enhanced Exchange Bias in La_0.6_Sr_0.4_MnO_3_/Orthorhombic YMnO_3_ Heterostructures

**DOI:** 10.1038/srep24568

**Published:** 2016-04-19

**Authors:** Dongxing Zheng, Chao Jin, Peng Li, Liyan Wang, Liefeng Feng, Wenbo Mi, Haili Bai

**Affiliations:** 1 Tianjin Key Laboratory of Low Dimensional Materials Physics and Preparation Technology, Institute of Advanced Materials Physics, Faculty of Science, Tianjin University, Tianjin 300072, China

## Abstract

The exchange bias in ferromagnetic/multiferroic heterostructures is usually considered to originate from interfacial coupling. In this work, an orbital reconstruction enhanced exchange bias was discovered. As La_0.6_Sr_0.4_MnO_3_ (LSMO) grown on YMnO_3_ (YMO) suffers a tensile strain (*a* > *c*), the doubly degenerate *e*_*g*_ orbital splits into high energy 3*z*^*2*^ − *r*^*2*^ and low energy *x*^*2*^ − *y*^*2*^ orbitals, which makes electrons occupy the localized *x*^*2*^ − *y*^*2*^ orbital and leads to the formation of antiferromagnetic phase in LSMO. The orbital reconstruction induced antiferromagnetic phase enhances the exchange bias in the LSMO/YMO heterostructures, lightening an effective way for electric-field modulated magnetic moments in multiferroic magnetoelectric devices.

## Introduction

The multiferroic (MF) heterostructures which integrates the ferromagnetic and ferroelectric orders together offer an effective route for new generation spintronic and optoelectronic devices^1^,^2^,^3^. Among these artificial structures, the exchange bias (EB) as a link to bridge the gap of the electric or magnetic field modulated magnetization has been widely studied^4^,^5^,^6^. Several mechanisms for the interfacial coupling in the heterostructures were reported, such as domain interaction^7^, interfacial magnetic inhomogeneity, interfacial superexchange coupling^8^,^9^,^10^ and the formation of interfacial ferromagnetic phase^11^,^12^,^13^. Considerable efforts focus on the interfacial coupling, whereas the intrinsic properties inside the ferromagnetic (FM) layer have not yet been taken into account so far. For example, in the strong correlated systems, like La_1−*x*_Sr_*x*_MnO_3_, the substrate strain or electric field can tailor their magnetic properties to several antiferromagnetic (AF) structures^14^,^15^.

To investigate the physical picture of EB in FM/MF heterostructures, the multiferroic orthorhombic YMnO_3_ (YMO) with E-type antiferromagnetic order was incorporated with the double-exchange ferromagnetic (FM) La_0.6_Sr_0.4_MnO_3_ (LSMO) and they were grown in different sequences. The reasons that we used YMO and LSMO in the heterostructures were based on the following considerations. Firstly, both of them are manganites, a strong exchange coupling can be expected due to the Mn^3+^–O^2−^–Mn^4+^ double exchange interaction at the interface. Secondly, both the orthorhombic YMO and LSMO films can be synthesized under similar conditions by applying proper substrate strain, so LSMO and YMO based heterostructures with different growth orders can then be achieved. Finally, the symmetric exchange striction mode^16^ induced polarization in YMO is not only larger than that of the others in orthorhombic RMnO_3_ systems, but also makes the magnetic field modulated polarization feasible, which will provide new perspectives to exploit the exchange coupling in multiferroic heterostructures^17^,^18^.

### Experiment and calculation details

By strain engineering, the YMO/LSMO and LSMO/YMO heterostructures with different lattice orientations on SrTiO_3_ (STO) single crystal substrates were fabricated by radio-frequency magnetron sputtering. The YMO and LSMO layer thicknesses were ∼50 and ∼12 nm, respectively. Details for the epitaxial growth of YMO and LSMO layers were referred to our previous work^19^. The lattice structures were analyzed by reciprocal space mapping (Supplementary Fig. S2) and transmission electron microscopy (TEM). Magnetic properties were measured using a Quantum Design magnetic property measurement system (SQUID-VSM). We also carried out the first-principles calculations based on the density-functional theory (DFT) and the projector augmented wave method as implemented in Vienna Ab initio Simulation Package code^20^,^21^ to study the magnetic properties of the heterostructures. For the exchange and correlation functional, we used the Perdew-Burke-Ernzerhof spin-polarized generalized gradient approximation^22^. The plane-wave basis set was converged using a 500 eV energy cutoff. A Γ-centered 6 × 3 × 1 *k*-mesh was used for the Brillouin-zone integrations.

## Results and Discussion

**Figure 1:**
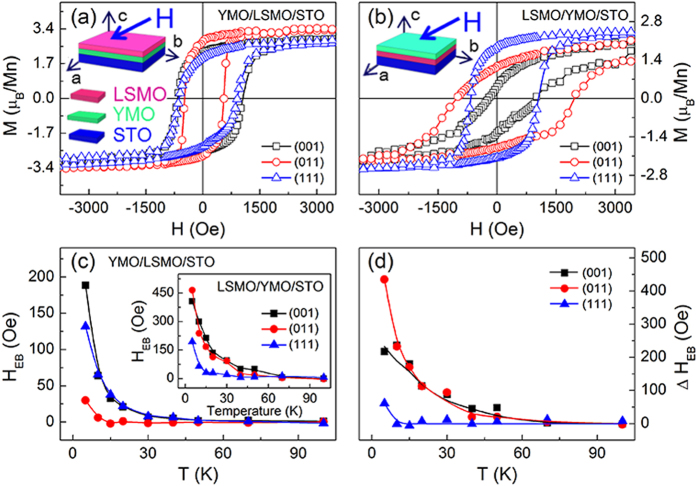
*M*-*H* curves of the (**a**) YMO/LSMO/STO and (**b**) LSMO/YMO/STO heterostructures with different lattice orientations. (**c**) Temperature-dependent EB field in the YMO/LSMO/STO heterostructures with different lattice orientations. The inset shows the temperature-dependent EB field in the LSMO/YMO/STO heterostructures. (**d**) Temperature-dependent Δ*H*_EB_ with different lattice orientations.

**Figure 2:**
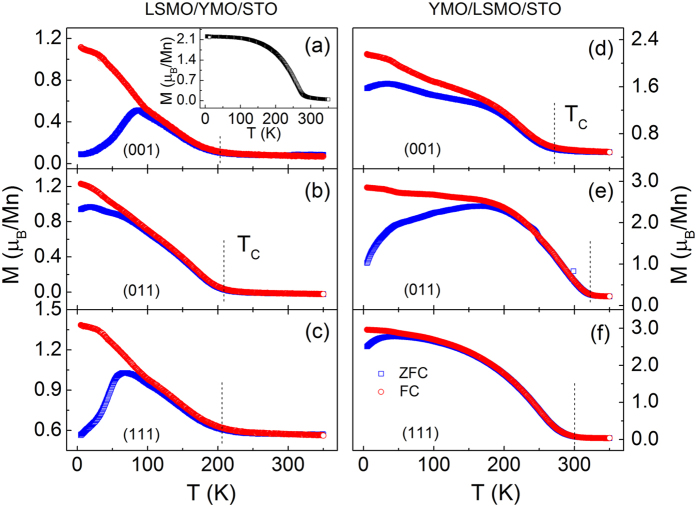
ZFC and FC curves of the LSMO/YMO/STO and YMO/LSMO/STO heterostructures with (**a,d**) (001), (**b,e**) (011) and (**c,f**) (111) orientations. The inset gives the *M*-*T* curve of the LSMO(001) single layer.

Our previous study on the magnetic properties of the YMO/LSMO/STO heterostructures with different lattice orientations showed that EB strongly depends on the lattice orientations with different Mn^3+^–O^2−^–Mn^4+^ bond angles at the interface^19^. Herewith, after the field cooling from 350 to 5 K with an in-plane magnetic field of 1 T, the hysteresis loops were measured. In Fig. 1(a), similar results are observed in the YMO/LSMO/STO heterostructures with the strongest EB in YMO/LSMO(001)/STO orientated sample. However, as shown in Fig. 1(b), the magnetic properties of the LSMO/YMO/STO are quite different from those in the YMO/LSMO/STO heterostructures. Several major characteristics are: (1) the saturation magnetization of ~2.6 μ_B_/Mn in the LSMO/YMO/STO heterostructures is much smaller than ~3.2 μ_B_/Mn in the YMO/LSMO/STO heterostructures; (2) the coercivity and EB field in Fig. 1(b) are larger than those in Fig. 1(a), indicating an enhanced magnetic anisotropy; (3) the magnetization in the YMO/LSMO/STO heterostructures is easier to be saturated than that in the LSMO/YMO/STO heterostructures. Why such significant differences occur to both series samples? To get more information about the magnetic properties of the two series samples, the temperature dependent EB with different lattice orientations are shown in Fig. 1(c). An obvious EB is observed in the YMO/LSMO/STO heterostructures with (001) orientation, on contrary to the weaker EB in the (011)-oriented YMO/LSMO/STO. With the increase of temperature, the EB field decreases and finally disappears around 45 K that is the Néel temperature of YMO (Supplementary Fig. S3)^23^,^24^. The lattice-orientation dependent EB can be attributed to the difference of the interfacial Mn^3+^–O^2−^–Mn^4+^ bond angle which leads to the different strength of interfacial coupling in the heterostructures^19^. As a comparison, the temperature dependent EB of the LSMO/YMO/STO heterostructures is also shown in the inset of Fig. 1(c). The EB fields of the LSMO/YMO/STO heterostructures are much larger than those of the YMO/LSMO/STO heterostructures, and do not disappear even above the Néel temperature of YMO. To further clarify the temperature-dependent EB, the temperature-dependent EB field Δ*H*_EB_ (Δ*H*_EB_ = *H*_EB(LSMO/YMO)_−*H*_EB(YMO/LSMO)_) is given in Fig. 1(d), indicating that some other factors also contribute to the EB in the heterostructures besides the interfacial coupling. Generally, EB is induced by the pinning effect of AFM phase and disappears above its Néel temperature^25^,^26^. However, in the LSMO/YMO/STO heterostructures, EB still appears around 60 K that is above the Néel temperature of YMO (45 K). Therefore, an AFM phase with a higher Néel temperature may exist in the LSMO/YMO/STO heterostructures. To trace the AFM phase, the zero-field cooling (ZFC) and field cooling (FC) curves of the LSMO/YMO/STO and YMO/LSMO/STO heterostructures with different lattice orientations were measured, as shown in Fig. 2. Herewith, the samples were cooled down from 350 to 5 K under a zero magnetic field. Then, a 200 Oe field was applied to collect the magnetization signal with increasing temperature. After that, a 200 Oe field was applied and cooled down the sample again from 350 to 5 K. In the FC measurement, the magnetization of all the samples decreases with increasing temperature, and approaches to a constant value at a certain temperature. The transition temperature is the FM Curie temperature. A bifurcation between the ZFC and FC curves are distinct, which indicates the phase separation in the LSMO layer^27^ or the magnetic frustration at interfaces between LSMO and YMO^28^. The bifurcation in the LSMO/YMO/STO heterostructures are much larger than that of YMO/LSMO/STO except for (011) orientation. Given the enhanced EB field and suppressed magnetization in the YMO/LSMO/STO heterostructures, it is believed that the larger bifurcation results from phase separation in the LSMO layer and magnetic frustration at the interfaces. Furthermore, the smaller bifurcation in the LSMO(011)/YMO/STO heterostructures may be ascribed to the spontaneous EB effect which forms a magnetic easy axis related to the initial applied magnetic field^29^. The inset of Fig. 2(a) shows the *M-T* curve of the LSMO(001) single layer. The Curie temperature of ~280 K and magnetization of ~2.2 μ_B_/Mn measured under 200 Oe at 5 K are close to the YMO/LSMO(001)/STO heterostructure with the values of ~275 K and ~2.3 μ_B_/Mn. From the ZFC and FC curves, it is clear to see that not only the Curie temperature (~200 K) of the LSMO layers in the LSMO/YMO heterostructures are much lower than that (~300 K) of the LSMO layers in the YMO/LSMO/STO heterostructures, but also the magnetization of the LSMO/YMO/STO heterostructures is greatly suppressed. The reduction of magnetization probably originates from several factors, such as oxygen vacancies^30^, instabilities of Mn valence^31^, segregation^32^ or strain induced phase separation^33^. The only difference between the LSMO layers lies in the reverse growth. It is thus reasonable to speculate that the YMO layer may introduce a strain into the LSMO layer in the LSMO/YMO/STO samples due to the large lattice misfit of ~6%. Indeed, the strain not only results in the formation of AFM phase in the LSMO layer, but also induces a distortion of MnO_6_ octahedra that strongly suppresses the FM Curie temperature^34^.

**Figure 3:**
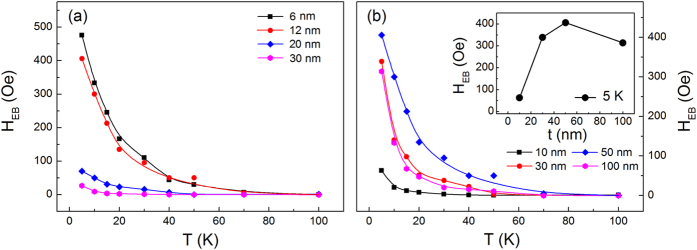
Temperature dependent EB field in the LSMO(001)/YMO/STO heterostructures with different (**a**) LSMO and (**b**) YMO thicknesses, the inset shows the YMO thickness dependent EB field at 5 K.

The LSMO and YMO thickness dependent EB in the LSMO(001)/YMO/STO heterostructures are shown in Fig. 3. For the EB induced by interfacial coupling, it is thickness dependent with the relation of *H*_EB_ = −*J*_EB_/*μ*_*o*_*M*_*F*_*t*_*F*_^26^ where *J*_EB_ is the interfacial exchange coupling energy, *t*_*F*_ and *M*_*F*_ the thickness and saturation magnetization of the ferromagnetic layer. As shown in Fig. 3(a), similar to the situation in the YMO/LSMO/STO heterostructures^19^, the EB also decreases with the increasing thickness of LSMO layer. This behavior could be understood on the following points. On one hand, the EB is thickness dependent in ferromagnetic/antiferromagnetic heterostructures. On the other hand, the strain is thickness dependent. With the increasing thickness of LSMO layer, the strain decreases and the content of AFM order becomes lower.

The YMO thickness dependence of EB is shown in Fig. 3(b). We fixed LSMO thickness at ~12 nm and varied YMO thickness with 10, 30, 50 and 100 nm. The EB first increases and then decreases. This trend is consistent with the study of antiferromagnetic thickness dependent EB in ferromagnetic/antiferromagnetic systems, which is ascribing to the thickness dependent domain wall energy^35^. In addition, the strain effects also contribute to this trend. When the thickness of YMO layer is ~10 nm, the strain referred to the STO substrate is not fully relaxed, and the growth of LSMO is strongly influenced by the STO substrate. In this case, the LSMO layer suffers weak strain from YMO. With the increasing thickness of YMO layer, the strain induced by YMO layer increases, which enhances the EB. However, further increase the YMO layer thickness to ~100 nm, the EB decreases. This decreasing trend of EB is due to the instability of orthorhombic YMO. The orthorhombic structure is a metastable state of YMO which can only be synthesized by applying substrate strain or under high pressure^18^. With the thickness of ~100 nm, the substrate strain is released in YMO layer and a multi-orientations surface may form at the surface. The multi-orientations surface not only decreases the YMO induced strain in the LSMO layer, but also weakens the interfacial exchange coupling strength which is strongest in the (001) orientation^19^. Thus a reduced EB is discovered.

**Figure 4:**
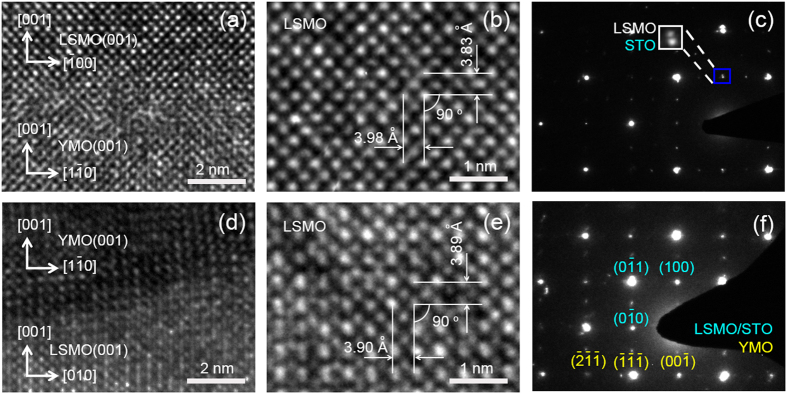
HRTEM images of the (**a,d**) LSMO(001)/YMO and YMO/LSMO(001) interfaces, corresponding to (**b,e**) LSMO layers and (**c,f**) SAED patterns of the LSMO/YMO/STO and YMO/LSMO/STO heterostructures.

To characterize the effect of strain on magnetic properties, the high resolution transmission electron microscopy (HRTEM) was employed to investigate the microstructure of the LSMO/YMO/STO and YMO/LSMO/STO heterostructures. Figure 4(a,d) show the interfacial structure of the LSMO(001)/YMO and YMO/LSMO(001) heterostructures. The lattice in the LSMO and YMO layers arranges orderly even though the LSMO(001)/YMO heterostructure exhibits a diffusion interface. The diffusion interface may come from the large lattice misfit between YMO and LSMO. In the YMO/LSMO(001) heterostructures, a well-defined interface with lattice ordered in nice pattern is visible. Figure 4(b) shows a cross-sectional view of the LSMO layer in the LSMO(001)/YMO heterostructures. There are two sets of lattice planes (001) and (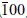) with the lattice plane distance of 3.83 Å and 3.98 Å. In Fig. 4(e), as the LSMO layer was directly grown on STO, the small lattice misfit of 0.6% gives the close plane distance of 3.90 Å and 3.89 Å. Upon this comparison, we confirm that the LSMO grown on YMO suffers tensile strain. The selected area electron diffraction (SAED) patterns of the heterostructures are shown in Fig. 4(c,f). Different from the overlap of the diffraction patterns in the YMO/LSMO(001) heterostructures, the diffraction patterns of the LSMO and STO layers in the LSMO(001)/YMO heterostructures separate from each other as indicated in the inset of Fig. 4(c), showing that the LSMO layer suffers a strain from the YMO. With a further analysis on the SAED patterns, we found that the lattice zone axes of LSMO and YMO are [010] and [110] in the LSMO(001)/YMO heterostructures and [100] and [110] in the YMO/LSMO(001) heterostructures. So the epitaxial relationships are YMO(001)[110]||LSMO(001)[010] and LSMO(001)[100]|| YMO(001)[110].

Together with the analyses of reciprocal space mappings, HRTEM images and SAED patterns, the lattice parameters of YMO and LSMO are listed in Table 1. The in plane lattice parameters of YMO in LSMO/YMO/STO and YMO/LSMO/STO are 5.14 Å and 4.97 Å, respectively, which is smaller than the bulk value of 5.24 Å^36^, indicating a strain applied by the STO substrate and LSMO layer. In the LSMO/YMO/STO heterostructures, the in-plane lattice parameters *a* and *b* are 3.98(2) Å and 3.75(2) Å. This strongly indicates a rectangular growth of LSMO on the YMO layer. Compared to the bulk value of 3.87 Å, the LSMO layer elongates in *a*,*b* plane and shrinks along *c*-axis.

**Figure 5:**
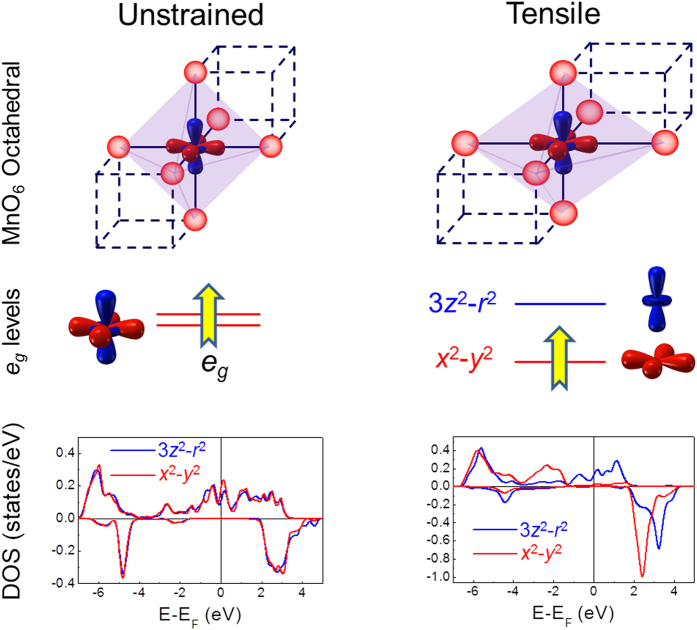
Orbital reconstruction of LSMO layers in the heterostructures, top panel: representation of the MnO_6_ octahedral distortions as a function of strain; middle panel: orbital reconstruction of the *e*_*g*_ orbitals of Mn ions; bottom panel: calculated density states of Mn ions.

How does the tensile strain affect the EB effect? In manganites, the key parameter governing the physical properties is the Mn^3+^ 3*d*^4^ orbital configuration. In spherical symmetry, the 3*d* orbitals are five-folds degenerate. For the unstrained perovskite manganites, as shown in the top panel of Fig. 5, the Mn^3+^ ion is surrounded by six O^2−^ with the octahedral symmetry. The wave function of *e*_*g*_ orbital stretches along the <100> axes on which the nearest neighbor O^2−^ is located, so that the *e*_*g*_ orbital is increased with doubly degenerate because of the strong Coulomb interaction between the negatively charged electron and the O^2−^. When the MnO_6_ octahedral suffers a tensile (*a* > *c*) strain, the Coulomb interaction is suppressed and the *x*^2^ − *y*^2^ orbital shifts to the low energy orbital. Thus the *e*_*g*_ electron tends to occupy it and becomes localized. Due to the orbital reconstruction of the *e*_*g*_ electron, the double exchange interaction is suppressed and the layer-typed antiferromagnetic order is formed^16^,^37^,^38^,^39^.

The results were further confirmed by the DFT calculation. The calculated densities of states (DOS) of Mn ions are shown in the bottom panel of Fig. 5. For the unstrained single layer of LSMO, the DOS of 3*z*^2^ − *r*^2^ and *x*^2^ − *y*^2^ orbitals exhibits a similar occupancy, indicating a doubly degenerate orbital. However, for the LSMO grown on the YMO layer, the tensile strain shifts the *x*^2^ − *y*^2^ orbital to the low energy level, leading to a localized and splited *e*_*g*_ orbital.

Therefore, the AFM phase was introduced into the LSMO layers by tensile strain induced orbital reconstruction when they were grown on YMO. The coupling of orbital reconstruction induced AFM order with the intrinsic FM order leads to an enhanced magnetic anisotropy, suppressed saturation magnetization, reduced FM Curie temperature and enhanced EB. Similarly, for the LSMO(011)/YMO/STO and LSMO(111)/YMO/STO heterostructures, the strain will also bring AFM order to this system and contribute to the EB.

## Conclusion

An enhanced EB in the LSMO/YMO/STO as compared to the YMO/LSMO/STO heterostructures was discovered, which can be ascribed to the strain induced orbital reconstruction. Consistent with the first principle calculation results, when the LSMO layer suffers a tensile strain from the YMO layer, the *x*^2^ − *y*^2^ orbital of LSMO shifts to the low energy level, and the *e*_*g*_ orbital was splited. The *e*_*g*_ electron occupies the low energy *x*^2^ − *y*^2^ orbital and becomes localized, thus an AFM order is formed. The coupling of FM order with the formed AFM order enhances the magnetic anisotropy and EB effect.

## Additional Information

**How to cite this article**: Zheng, D. *et al*. Orbital Reconstruction Enhanced Exchange Bias in La_0.6_Sr_0.4_MnO_3_/Orthorhombic YMnO_3_ Heterostructures. *Sci. Rep.* **6**, 24568; doi: 10.1038/srep24568 (2016).

## Supplementary Material

Supplementary Information

## Figures and Tables

**Table 1:** Lattice constants of the YMO and LSMO layers grown on STO (001) in the heterostructures.

Samples		*a*(Å)	*b*(Å)	*c*(Å)
LSMO/YMO	LSMO	3.98 (2)	3.75 (2)	3.83 (2)
	YMO	5.14 (2)		7.91 (2)
YMO/LSMO	LSMO	3.90 (2)	3.90 (2)	3.89 (2)
	YMO	4.97 (2)		7.97 (2)
