# Supplementary material for: Orbital Reconstruction Enhanced Exchange Bias in La0.6Sr0.4MnO3/Orthorhombic YMnO3 Heterostructures
Source: Sci Rep. 2016 Apr 19;6:24568. doi: 10.1038/srep24568 (PMC4836304; doi:10.1038/srep24568)
Supplement: Supplementary Information [file srep24568-s1.pdf]

Supplementary Information

Orbital Reconstruction Enhanced Exchange Bias in $La_{0.6}Sr_{0.4}MnO_3$/Orthorhombic $YMnO_3$ Heterostructures

Dongxing Zheng, Chao Jin, Peng Li, Liyan Wang, Liefeng Feng, Wenbo Mi, and Haili Bai*

Tianjin Key Laboratory of Low Dimensional Materials Physics and Preparation Technology, Institute of Advanced Materials Physics, Faculty of Science, Tianjin University, Tianjin 300072, China

*E–mail: baihaili@tju.edu.cn

*Author to whom all correspondence should be addressed.

E-mail: baihaili@tju.edu.cn

The schematics of structural growth relation in each case are shown in Fig. S1. According to the value given in Tab. 1, when the LSMO is directly deposited on the STO substrate, the close lattice parameters make it suffers weak strain from the substrate. As shown in Fig. S1(a), the YMO is growing on the diagonal direction of LSMO with the relation YMO(100)//LSMO(110). In Fig. S1(d), for the inverse growth, the diagonal direction of LSMO is parallel to YMO(100). The other cases are also shown in Fig. S1.

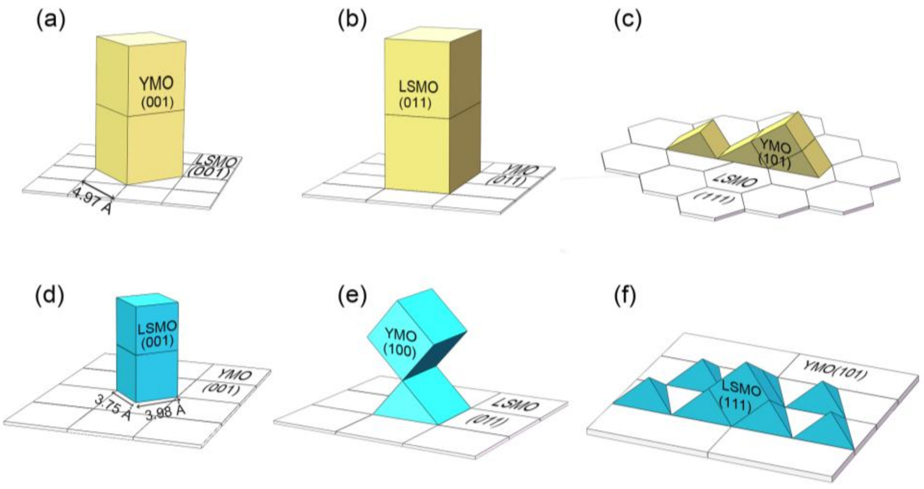

Figure S1. Schematics for the structural growth relation (a) YMO(001)/LSMO(001) on STO(001); (b) YMO(100)/LSMO(011) on STO(011); (c) YMO(101)/LSMO(111) on STO(111); (d) LSMO(001)/YMO(001) on STO(001); (e) LSMO(011)/YMO(100) on STO(011); and (f) LSMO(111)/YMO(101) on STO(111).

The reciprocal space mapping data collected around the STO (103) for the LSMO/YMO/STO and YMO/LSMO/STO heterostructures are shown in Fig. S2. Only the STO (103) and YMO (106) diffraction patterns are visible. The in plane lattice parameters of YMO in LSMO/YMO/STO and YMO/LSMO/STO are 5.14 Å and 4.97 Å, respectively, which are smaller than the bulk value of 5.24 Å[1], indicating a strain induced growth of YMO on the STO substrate and LSMO layer. When the YMO is growing on the LSMO layer, the lattice reduces to 4.97 Å. This is due to the smaller lattice parameter of LSMO (3.90 Å) as compared to that of STO (3.905 Å) substrate. As the reciprocal space mapping is very sensitive to layer thickness, the diffraction pattern of the LSMO layer with small thickness of 12 nm is not observed.

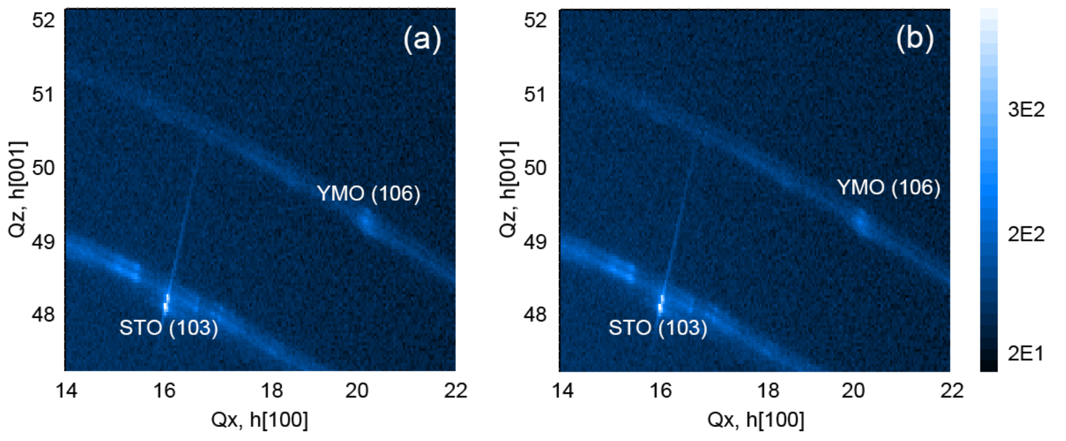

Figure S2. Asymmetric reciprocal space mapping of (a) LSMO/YMO/STO and (b) YMO/LSMO/STO heterostructures.

In Fig. S3, it can be clearly seen that the χ^{-1} deviated from the dash line at ~45 K, which means a transition of magnetic properties from paramagnet to antiferromagnet. So the Néel temperature of the YMO film can thus be decided to be ~45 K. This is consistent with other group's work.[2, 3]

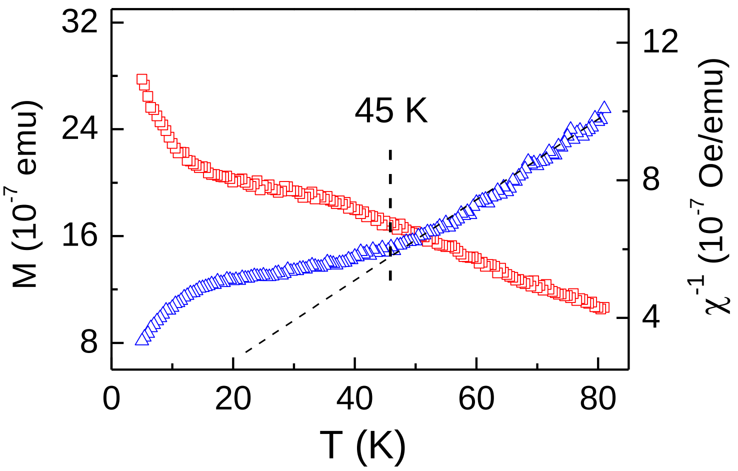

Figure S3. Temperature-dependent magnetization and inverse susceptibility of the YMO single layer.

The *M-H* loop for LSMO(001) single layer is shown in Fig. S4, almost no shift is discovered. Moreover, with the increase of temperature, the H_{EB} of LSMO layer is close to zero, which is negligible as compare to those of LSMO(001)/YMO/STO and YMO/LSMO(001)/STO.

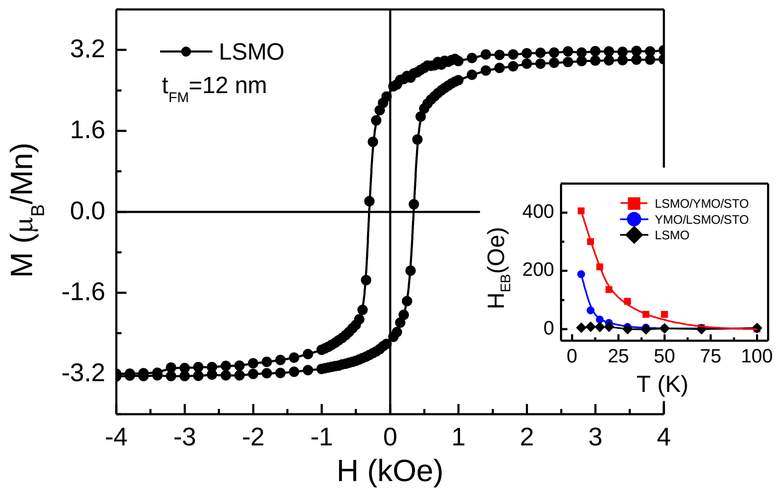

Figure S4. *M-H* loop of the LSMO(001) single layer measured at the same conditions of the heterostructures. The inset shows the temperature dependence of exchange bias in LSMO(001)/YMO/STO, YMO/LSMO(001)/STO and LSMO(001) single layer.

The spin configuration in different heterostructures is shown in Fig. S5. For the single LSMO and YMO layers, their spin configurations are ferromagnetic and E-type antiferromagnetic with a zig-zag chain (↑↑↓↓), respectively. As we mentioned in this report, the EB is induced by interfacial coupling and orbital reconstruction in the LSMO/YMO/STO heterostructures, while in the YMO/LSMO/STO heterostructures, the exchange bias is mainly dominated by interfacial coupling. In Fig. S5, for the YMO/LSMO/STO heterostructures, the interfacial coupling originates from the double exchange interaction between the Mn^{3+}–O^{2-}–Mn^{4+} bond, which helps to form a ferromagnetic order at the interface region at YMO side.[4-7] However, in the LSMO/YMO/STO heterostructures, the LSMO layer suffers tensile strain, it supports the occupancy of e_g electron at x^2-y^2 orbital and left the $3z^2$-r^2 orbital vacant. Thus the inner plane double exchange interaction is enhanced while the inter plane double exchange interaction is weakened. As the inter plane double exchange interaction is weakened, the super exchange interaction between t_{2g} electron dominates the inter plane coupling, and an inter plane antiferromagnetic coupling is thus formed. This effect supports the formation of A-type antiferromagnetic order in the LSMO layer. At the interface region, the interfacial coupling between LSMO and YMO is super exchange interaction as the e_g electron is localized at the x^2-y^2 orbital.[8] Due to the strong inner plane ferromagnetic order of LSMO atomic layer at the interface, the YMO atomic layer turns into the ferromagnetic order at the interface region. The spin configuration of the LSMO/YMO/STO heterostructures is shown in right panel of Fig. S5.

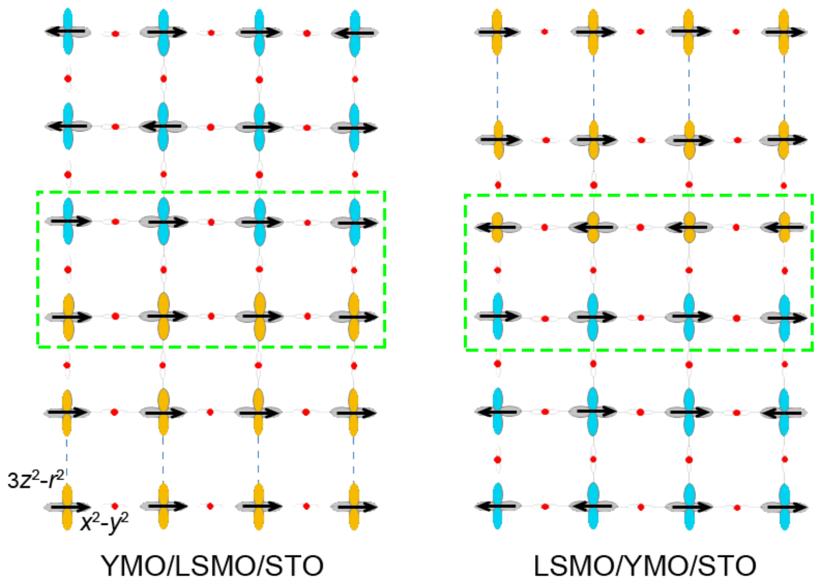

Figure S5. Schematic of spin configurations in the YMO/LSMO(001)/STO and LSMO(001)/YMO/STO heterostructures. The arrows indicate the spin orientation in Mn cations and the Mn *d* orbitals are drawn in orange (LSMO) and blue (YMO) for $3z^2$-r^2 and grey for x^2-y^2. The lobes of the *p* orbitals are shown around the oxygen atoms (red).

References

1. Munoz, A. et al. The magnetic structure of $YMnO_3$ perovskite revisited. *J. Phys. Condens. Matter.* **14**, 3285 (2002).
2. Lorenz, B., Wang, Y.Q., Sun, Y.Y., & Chu, C.W. Large magnetodielectric effects in orthorhombic $HoMnO_3$ and $YMnO_3$. *Phys. Rev. B.* **70**, 212412 (2004).
3. Nakamura, M., Tokunaga, Y., Kawasaki, M., & Tokura, Y. Multiferroicity in an orthorhombic $YMnO_3$ single-crystal film. *Appl. Phys. Lett.* **98**, 082902 (2011).
4. Paul, A., Zandalazini, C., Esquinazi, P., Autieri, C., Sanyal, B., Korelis, P. & Böni, P. Structural, electronic and magnetic properties of $YMnO_3/La_{0.7}Sr_{0.3}MnO_3$ heterostructures. *J. Appl. Crystallogr.* **47**, 1054−1064 (2014).
5. Zheng, D. X., Gong, J. L., Jin, C., Li, P. & Bai, H. L. Crystal orientation modulated exchange bias in orthorhombic-$YMnO_3$ /$La_{0.6}Sr_{0.4}MnO_3$ multiferroic heterostructures. *ACS Appl. Mater. Inter.* **7**, 14758 (2015).
6. Autieri, C. & Sanyal, B. Unusual ferromagnetic $YMnO_3$ phase in $YMnO_3/La_{2/3}Sr_{1/3}MnO_3$ heterostructures. *New J. Phys.* **16**, 113031 (2014).
7. Zandalazini, C., Esquinazi, P., Bridoux, G., Barzola-Quiquia, J., Ohldag, H. & Arenholz, E. Uncompensated magnetization and exchange−Bias field in $La_{0.7}Sr_{0.3}MnO_3/YMnO_3$ bilayers: the Influence of the ferromagnetic layer. *J. Magn. Magn. Mater.* **323**, 2892−2898 (2011).
8. Yu, P. *et al.* Interface ferromagnetism and orbital reconstruction in $BiFeO_3$-$La_{0.7}Sr_{0.3}MnO_3$ heterostructures. *Phys. Rev. Lett.* **105**, 027201 (2010).
